# Sex-specific associations of serum cotinine levels with depressive symptoms and sleep disorders in American adults: NHANES 2007-2014

**DOI:** 10.3389/fpsyt.2024.1434116

**Published:** 2024-12-10

**Authors:** Hongguang Yang, Yao Liu, Zhenhe Huang, Guifang Deng

**Affiliations:** ^1^ Department of Clinical Nutrition, Shenzhen Nanshan People’s Hospital, Shenzhen, Guangdong, China; ^2^ Geriatric Medicine Department, Shenzhen Nanshan People’s Hospital, Shenzhen, Guangdong, China

**Keywords:** tobacco smoke exposure, serum cotinine, human mental health, depression, sleep disorders, gender differences

## Abstract

**Background:**

Accumulating evidence have demonstrated that tobacco smoke exposure (TSE) causes damage to human mental issues. However, previous studies almost focus on the individual smoking exposure patterns and some inconsistent results are reported. Serum cotinine is a reliable and quantitative biomarker of TSE. This study aims to explore the association of serum cotinine with depression and sleep disorders and the potential gender differences.

**Methods:**

Data from the National Health and Nutrition Examination Survey (NHANES) 2007-2014 was used. Weighted multiple logistic regression methods, generalized additive models (GAM), and restricted cubic spline (RCS) models were used for association analyses. Moreover, gender-stratified analyses were conducted.

**Results:**

Of 12,599 individuals included in the final analysis, 1,295 had depression, 3245 had trouble sleeping and 1152 had diagnostic sleep disorders. After adjusting for potential covariates, linear relationship suggested higher serum cotinine levels were positively associated with risk of depression and sleep disorders, including self-reported trouble sleeping and diagnostic sleep disorders in the total sample and female participants, and serum cotinine levels were positively correlated with depression and trouble sleeping in male participants. Additionally, inverted L-shaped associations between serum cotinine and depression and sleep disorders were detected, and at the same cotinine level, females have a higher risk of experiencing depression and sleep disorders.

**Conclusions:**

In this study, higher serum cotinine increased the risk of depression and sleep disorders and there was stronger association in females than males. These findings provided novel evidence about how TSE affected the mental condition of the general US population.

## 1 Introduction

Accumulating epidemiological evidence has demonstrated a close relationship between tobacco smoke exposure (TSE) and various health damages and diseases, leading to an increasing global burden on society and economy (1, 2). It has been widely known that TSE increases the risk of lung cancer (3), respiratory diseases (3), cardiovascular diseases (4), reproductive and developmental diseases (5), and mental health issues (6). It is estimated that more than 200 million deaths have been caused by smoking tobacco use over the past 30 years and over eight million premature deaths and over 1 trillion dollars economic costs are attributable to TSE annually (7, 8).

Depression and sleep disorders are significant public health issues that seriously impair mental health and increase risks of various chronic diseases and mortality (6, 9, 10). Moreover, depression and sleep disorders exhibit a high degree of comorbidity, with sleep disorders potentially being both a consequence and a contributing factor to depression (11–13). Previous studies demonstrate that TSE is a main behavior risk of depression and sleep disorders (14). Smoking is even reported to have a causal effect on depression (15). Not only is the active smoking widely considered a risk factor for these mental health disorders, but secondhand smoke (SHS) has also been linked to mental health issues including depression, anxiety, and sleep disorders (6). Although majority of studies report that both active cigarette smoking and passive smoke exposure are found to be associated with sleep disturbance (16), inconsistent research results are presented. For example, Wang et al. reported an association between SHS and poor sleep quality in self-reported never-smokers in China (17), while a nationwide study using data from the National Health and Nutrition Examination Survey (NHANES) reported no significant associations between SHS and sleep disorders (18).

However, previous studies mainly evaluate TSE based on self-reported smoking status, and the definitions are inconsistent, introducing bias into the associations between TSE and research outcomes. Moreover, self-reported estimates of both active smoking and passive smoke exposure have been shown to be much lower than biomarker-determined exposure (19). Recent studies have sought to replace self-reported smoking status with serum cotinine levels as a more accurate and objective measure for assessing TSE (20, 21). Previous studies have explored the association between serum cotinine exposure and various health issues such as liver disease (22), Alzheimer’s disease (23), and diabetes (24, 25). However, research examining the association of cotinine levels with depression and sleep disorders remains scarce.

In this study, a cross-sectional study is conducted based on data from 4 cycles of NHANES 2007-2014. We aim to investigate the association of serum cotinine concentrations with depression and sleep disorders and to determine whether the gender differences in the associations exist.

## 2 Methods

### 2.1 Data source and study population

NHANES is a cross-sectional and nationwide survey conducted by the National Center for Health Statistics of the Centers for Disease Control and Prevention (CDC), aiming to assess the health and nutrition status of children and adults in the US. The survey is conducted every two years, and demographic data, dietary data, examination data, and laboratory data were collected. Complex, multistage, and probability sampling methods are used to obtain representative data of the general population in the United States. The relevant information of NHANES can be accessed at https://www.cdc.gov/nchs/nhanes/about_nhanes.htm.

**Figure 1:**
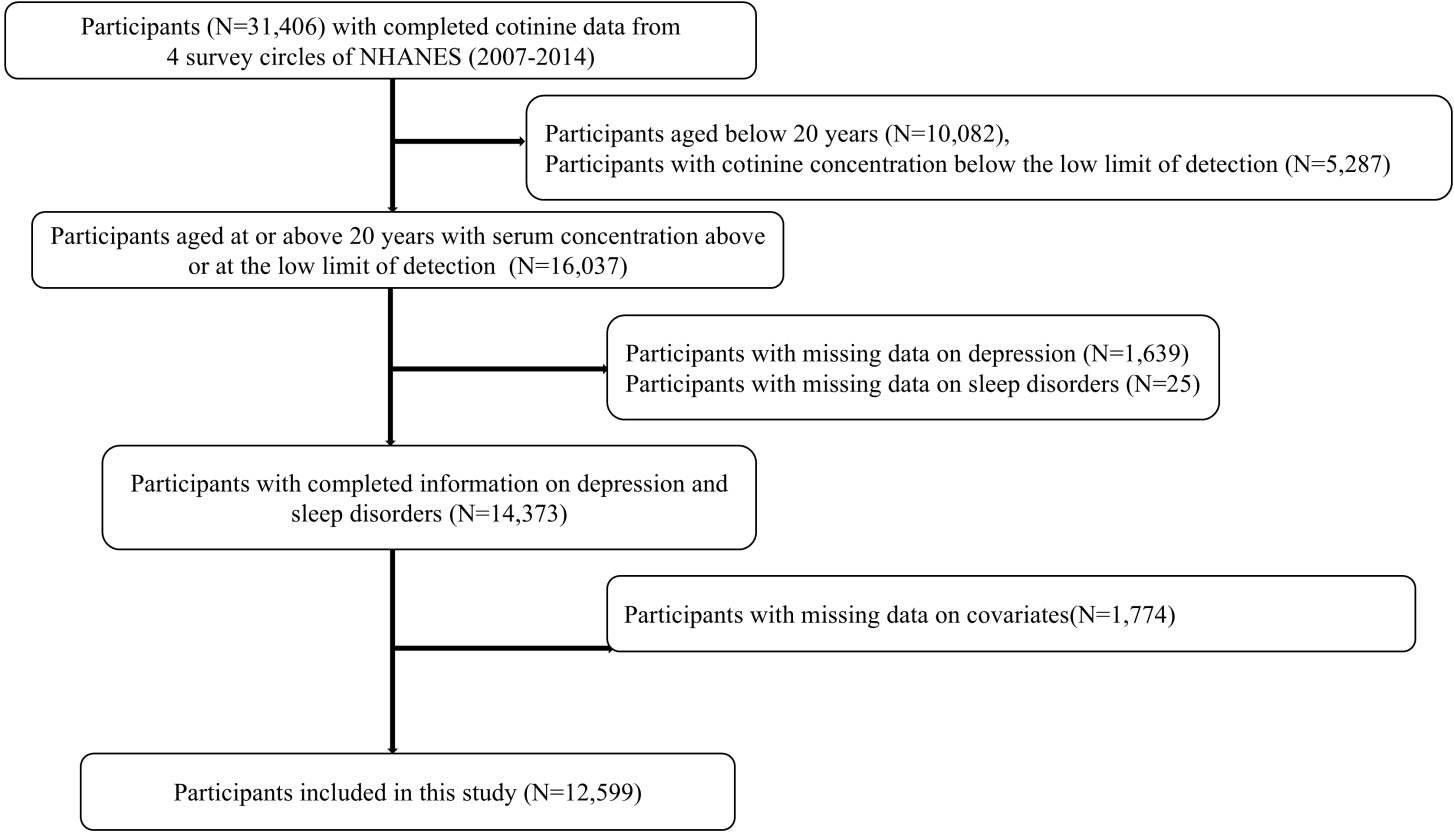
Flow chart population included in the analysis.

Data used in this study was from four cycles of datasets from NHANES (2007-2014). Among 31,406 participants with completed serum cotinine data from NHANES 2007-2014, we excluded 10,082 participants aged below 20 years, 5,287 participants with cotinine concentration below the low limit of detection, 1,639 participants with missing data on depression, 25 participants with missing data on sleep disorders, and 1,774 with missing data on covariates. Finally, a total of 12,599 participants were recruited in the study. Additional details of the study design, sampling, and exclusion procedures were illustrated in **Figure 1**.

### 2.2 Serum cotinine measurement

Serum cotinine is measured by an isotope-dilution high-performance liquid chromatography/atmospheric pressure chemical ionization tandem mass spectrometric (ID HPLC-APCI MS/MS) method. Briefly, the serum sample is spiked with methyl-D3-cotinine and methyl-D3-hydroxycotinine as internal standards. The cotinine was measured by m/z 80 product ion from the m/z 177 quasi-molecular ion. Analyte concentrations are derived from the area ratios of native-to-labeled compounds in the sample by comparisons to a standard curve. Details of the serum cotinine testing methods is available https://wwwn.cdc.gov/Nchs/Nhanes/2013-2014/COT_H.htm.

The lower limit of detection (LLOD) of serum cotinine was 0.015 ng/mL, and participants with serum cotinine concentrations below LLOD were excluded from this study referring to the previous study (21).

### 2.3 Assessment of depression and sleep disorders

The depressive symptoms were measured by the Patient Health Questionnaire-9 (PHQ-9), a good self-report instrument that assessed depression symptoms over the past 2 weeks. The PHQ-9 questionnaire contains nine items, in which response categories “not at all” “several days” “more than half the days” and “nearly every day” were given a point ranging from 0 to 3 and summing up a total scale range of 0 to 27. A total score ≥ 10 was considered as depression (13, 26, 27).

Sleep disorders were assessed by Sleep Disorder Questionnaire in NHANES. Participants were asked whether they have ever told a doctor or other health professional that they have trouble sleeping and whether they have been told by a doctor or other health professional that they have a sleep disorder. The participants who answered “Yes” were considered with trouble sleeping or diagnostic sleep disorder. The answers of “Do not know” and “Refused” were considered missing.

### 2.4 Covariates

Demographic characteristics and data on lifestyle and diabetes were collected through home interviews. The race was classified into four categories: non-Hispanic white, non-Hispanic black, Mexican American, and other or multiracial (28). Family income level was evaluated by family income-to-poverty ratio (FIPR) (≤1.3, 1.3–3.5, >3.5) (29). Educational levels were classified into four categories: less than high school graduated, high school graduated, some college or AA degree and college graduate or above. Body mass index (BMI) was calculated through dividing weight by square of height (kg/m^2^) and divided into three stratifications: ≤24.9, 25-29.9, and >30 kg/m^2^ (30). Drinking status was classified into two groups: no (those who had less than 12 alcohol drinks <12 times per year) and yes (those who had at least 12 alcohol drinks per year).

The physical activity was assessed by the Global Physical Activity Questionnaire (GPAQ), which included questions related to daily activities, leisure time activities, and sedentary activities. Physical activity levels were further categorized by metabolic equivalent (MET) scores using the recommended MET score for each activity type in NHANES. Finally, participants were divided into three groups: no physical activity group (0 MET-h/week), low intensity physical activity group (≤48 MET-h/wk) and high intensity physical activity group (> 48 MET-h/wk) (31, 32).

In addition, Healthy Eating Index-2015 (HEI-2015) score was calculated for each participant to assess diet quality based upon the Dietary Guidelines for Americans 2015–2020 (33).

Age and HEI-2015 scores were treated as continuous variables in subsequent analyses.

### 2.5 Statistical analysis

Analyses were conducted according to the guidelines for the analysis of NHANES data. Considering the complex survey design, an appropriate weight was used to provide nationally representative estimates and the weighted data was employed for subsequent analyses.

Serum cotinine concentrations were divided into four categories by quartiles in the weighted samples. The cut-off values were 0.031, 0.139, and 128.000 ng/mL, respectively. Descriptive information about demographic characteristics stratified by cotinine groups was represented as numbers (n) and percentages (%) for categorical variables and the mean and standard deviation (SD) or median and inter-quartile range (IQR) for continuous variables. Differences among groups were tested by using the Scott-Rao chi-square tests for categorical variables and ANOVA or Kruskal-Wallis tests for continuous variables.

The weighted logistic regression models were built to access the association between cotinine exposure and sleep disorders, in which the serum cotinine concentrations were employed as continuous variable and quartile categories (the first quartile was assigned as the reference group) exposure variables. Two models were used to explore the linear relationships. Model 1 was adjusted for age, gender and race; Model 2 was further adjusted for educational levels, FIPR, drinking status, physical activity, diabetes status, and HEI-2015 and added depression for sleep disorders (trouble sleeping and diagnostic sleep disorders) and trouble sleeping for depression, respectively. In addition, the ln-transformed serum cotinine concentrations were also used as continuous variables in the models.

Restricted cubic spline (RCS) and generalized additive model (GAM) were statistical approaches for fitting nonlinear relationships and were applied to analyze the nonlinear and dose-response relationships between serum cotinine concentrations and depression and sleep disorders. RCS is characterized by its linear restriction at the data range extremities, which helps in curbing overfitting, particularly during extrapolation. GAM extends beyond generalized linear models (GLM) by relaxing strict linearity assumptions, allowing for a variety of functions, including splines, local regression, or kernel functions, to model the relationships between variables. All the weighted models were adjusted for age, gender, race, educational levels, FIPR, drinking status, physical activity, diabetes status, and HEI-2015 and depression for sleep disorders (trouble sleeping and diagnostic sleep disorders) and trouble sleeping for depression, respectively. Generally, the effective degree of freedom (EDF) was obtained to estimate non-linearity, where EDF = 1 was considered as linearity and EDF > 1 was considered as non-linearity (34).

In addition, subgroups analyses were preformed stratified by gender as the gender differences were reported in previous studies. We constructed gender-stratified the smooth curve fitting methods and RCS analyses in subpopulations to estimate the nonlinear and dose-response relationships.

All statistical analyses were performed by using R software (version 4.2.2; R Core Team) and EmpowerStats (version 3.0). A two-sided P <0.05 considered statistically significant.

## 3 Results

### 3.1 Characteristics of the study population

The baseline characteristics of the study population stratified by serum cotinine concentrations (Q1: 0.015–0.031 ng/mL, Q2: 0.031–0.139 ng/mL, Q3: 0.139–128.000 ng/mL, Q4: 128.000-1820.000 ng/mL) were summarized in **Tables 1** and **2**. Of 12,599 participants included in the analysis, 6,611 were males, 1,295 had depression, 3245 had trouble sleeping and 1152 had diagnostic sleep disorders. As shown in **Tables 1** and **2**, age, gender, race, educational levels, physical activity, alcohol drinking, BMI levels, HEI-2015 and diabetes prevalence of participants in the four subgroups were summarized and significant differences were detected among the four subgroups. In addition, the prevalence of depression, trouble sleeping and diagnostic sleep disorder were found to increase in correlation with serum cotinine levels across quartiles.

**Table 1:** Baseline data of participants in groups stratified by serum cotinine concentrations.

	Total (N=12599)	Q1 (0.015-0.031) (N=3102)	Q2 (0.031-0.139) (N=3188)	Q3 (0.139-128.000) (N=3283)	Q4(128.000)-(N=3026)	p
**Age, year**	45.49 ± 16.45	52.01 ± 16.42	49.38 ± 17.29	44.02 ± 16.46	45.95 ± 14.39	<0.001
Gender, n (weighted %)						<0.001
Male	6611 (52.4)	1676 (55.0)	1583 (48.7)	1503 (45.7)	1226 (41.0)	
Female	5988 (47.6)	1426 (45.0)	1605 (51.3)	1780 (54.3)	1800 (59.0)	
Race, n (weighted %)						<0.001
Mexican American	2777 (12.6)	899 (15.0)	790 (14.5)	777 (15.2)	311 (5.7)	
Non-Hispanic White	5804 (68.2)	1333 (68.2)	1349 (65.7)	1347 (63.1)	1775 (76.0)	
Non-Hispanic Black	2920 (12.4)	510 (8.4)	934 (11.7)	934 (16.5)	768 (13.1)	
Other or multiracial	1908 (6.7)	360 (8.4)	225 (8.1)	225 (5.2)	172 (5.2)	
FIPR, n (weighted %)						<0.001
≤1.30	4521 (25.2)	757 (15.4)	946 (20.3)	1329 (29.5)	1489 (35.9)	
1.30-3.50	4608 (36.2)	1146 (33.8)	1234 (37.4)	1227 (37.6)	1001 (35.8)	
>3.50	3470 (38.6)	1199 (50.8)	1008 (42.4)	727 (32.9)	536 (28.3)	
Educational levels, n (weighted %)						<0.001
Less than high school graduated	3276 (18.5)	617 (11.9)	755 (15.8)	900 (19.8)	1004 (26.8)	
High school graduated	3145 (24.9)	598 (17.4)	726 (23.8)	882 (26.2)	939 (32.1)	
Some college or AA degree	3775 (32.8)	923 (32.1)	948 (32.1)	1038 (35.2)	866 (31.9)	
College graduate or above	2403 (23.7)	964 (38.6)	759 (28.2)	463 (18.8)	217 (9.2)	
Alcohol drinking, n (weighted %)						<0.001
No	3076 (19.6)	979 (24.9)	1000 (25.0)	669 (16.7)	428 (11.6)	
Yes	9523 (80.4)	2123 (75.1)	2188 (75.0)	2614 (83.3)	2598 (88.4)	
**Diabetes, n (weighted %)**	2247 (13.5)	629 (15.1)	612 (14.4)	554 (12.5)	452 (12.2)	0.01
BMI levels, n (weighted %)						<0.001
<25	3640 (29.9)	851 (29.4)	837 (26.6)	830 (26.7)	1122 (37.1)	
25 to <30	4118 (33.0)	1076 (34.8)	1047 (33.3)	1038 (32.7)	957 (31.1)	
≥30	4841 (37.1)	1175 (35.9)	1304 (40.1)	1415 (40.6)	947 (31.8)	
Physical activity, n (weighted %)						<0.001
No	3240 (22.0)	767 (21.2)	823 (20.9)	822 (21.4)	828 (24.3)	
Low	5231 (43.2)	1501 (50.4)	1377 (44.8)	1298 (42.3)	1055 (35.2)	
High	4128 (34.8)	834 (28.4)	988 (34.3)	1163 (36.3)	1143 (40.5)	
**HEI-2015**	52.09 ± 13.08	56.48 ± 13.15	54.24 ± 13.21	50.57 ± 12.10	47.04 ± 11.76	<0.001

Sampling weights were applied for calculation of demographic descriptive statistics. The p value was calculated after weighting.

FIPR, family income-to-poverty index; BMI, body mass index; HEI-2015, healthy eating index 2015.

**Table 2:** Information about depression and sleep disorders in different groups stratified by serum cotinine concentrations.

	Total(N=12599)	Q1(N=3102)	Q2(N=3188)	Q3(N=3283)	Q4(N=3026)	*p*
Depression, n (weighted %)						<0.001
No	12304 (91.0)	2198 (95.2)	2928 (93.4)	2926 (90.4)	2532 (85.1)	
Yes	295 (9.0)	184 (4.8)	260 (6.6)	357 (9.6)	494 (14.9)	
Trouble sleeping, n (weighted %)						<0.001
No	9354 (73.0)	2394 (75.8)	2478 (76.7)	2415 (72.1)	2058 (67.3)	
Yes	3245 (27.0)	708 (24.2)	710 (23.3)	868 (27.9)	968 (32.7)	
Diagnostic sleep disorder, n (weighted %)						0.007
No	11447 (90.7)	2840 (91.4)	2917 (91.5)	2993 (91.1)	2697 (88.7)	
Yes	1152 (9.3)	262 (8.6)	271 (8.5)	290 (8.9)	329 (11.3)	

Sampling weights were applied for calculation of demographic descriptive statistics. The p value was calculated after weighting.

As shown in **Supplementary Table S1** in gender-stratified groups, the median (IQR) of serum cotinine concentrations were 0.139 (0.031, 128.000) ng/mL in total participants, 0.269 (0.036, 166.000) ng/mL in male participants, and 0.093 (0.028, 73.300) ng/mL in female participants, respectively. Female participants had lower serum cotinine concentrations and higher prevalence of depression and trouble sleeping.

### 3.2 The linear relationship analysis between serum cotinine and depression and sleep disorders

The linear relationships between serum cotinine and depression and sleep disorders were performed by weighted binary logistic regression models and the results were presented in **Table 3**. After adjusting for potential covariables, serum cotinine concentrations showed significant association with depression, trouble sleeping and diagnostic sleep disorder. Compared with Q1 of serum cotinine concentrations, the ORs (quartile, 95%CIs) of depression were 1.59 (Q3, 1.28-1.98) and 2.39 (Q4, 1.81-3.17); the ORs (quartile, 95%CIs) of trouble sleeping were 1.31 (Q3, 1.09-1.58) and 1.46 (Q4, 1.21-1.75); and the OR (quartile, 95%CIs) of diagnostic sleep disorder was 1.40 (Q4, 1.13-1.75).

**Table 3:** The relationship between serum cotinine and depression and sleep disorders.

		Model 1		Model 2	
		OR (95%CI)	*p*	OR (95%CI)	*p*
Depression					
	continuous	1.14 (1.13,1.16)	<0.001	1.08 (1.06,1.10)	<0.001
	Q1	ref.		ref.	
	Q2	1.51 (1.15,1.97)	0.003	1.34 (1.00,1.79)	0.052
	Q3	2.36 (1.91,2.92)	<0.001	1.59 (1.28,1.98)	<0.001
	Q4	4.27 (3.40,5.36)	<0.001	2.39 (1.81,3.17)	<0.001
	P for trend	<0.001		<0.001	
Trouble sleeping					
	Continuous	1.06 (1.05,1.08)	<0.001	1.05 (1.03,1.07)	<0.001
	Q1	ref.		ref.	
	Q2	1.03 (0.89,1.18)	0.682	0.98 (0.84,1.13)	0.77
	Q3	1.46 (1.23,1.74)	<0.001	1.31 (1.09,1.58)	0.005
	Q4	1.72 (1.46,2.03)	<0.001	1.46 (1.21,1.75)	<0.001
	P for trend	<0.001		<0.001	
Diagnostic sleep disorder					
	continuous	1.05 (1.02,1.07)	<0.001	1.05 (1.02,1.07)	<0.001
	Q1	ref.		ref.	
	Q2	1.03 (0.83,1.29)	0.761	0.97 (0.78,1.22)	0.808
	Q3	1.19 (0.95,1.50)	0.126	1.09 (0.85,1.39)	0.503
	Q4	1.46 (1.21,1.77)	<0.001	1.40 (1.13,1.75)	0.003
	P for trend	<0.001		<0.001	

Model 1 was adjusted by age, gender, race.

Model 2, for depression, was further adjusted by FIPR, educational level, physical activity, alcohol drinking, diabetes, BMI levels, HEI-2015 and trouble sleeping; for trouble sleeping and diagnostic sleep disorder was further adjusted by age, gender, race, FIPR, educational level, physical activity, alcohol drinking, diabetes, BMI levels, HEI-2015 and depression.

When performed ln-transformed serum cotinine concentrations as continuous variables in the weighted logistic regression models, the similar positive associations of serum cotinine concentrations and depression, trouble sleeping and diagnostic sleep disorder were remained. The multivariate-adjusted OR (95%CIs) reached 1.08 (1.06-1.10) for depression, 1.05 (1.03-1.07) for trouble sleeping, and 1.05 (1.02-1.07) for diagnostic sleep disorder.

### 3.3 The nonlinear relationships between serum cotinine and depression and sleep disorders

**Figure 2:**
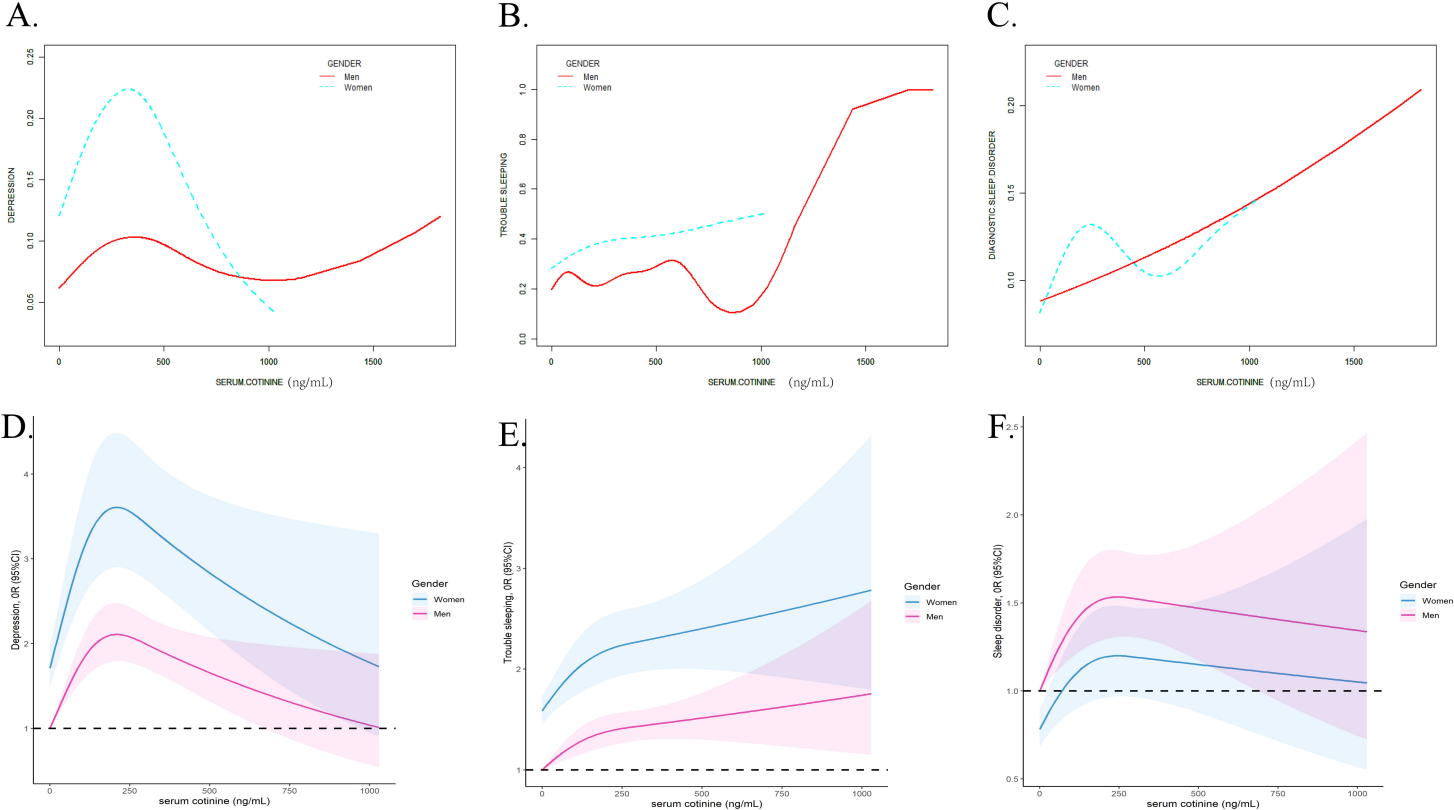
The non-linear association analyzed by the smooth curve fitting method between serum cotinine and depression **(A)**, trouble sleeping **(B)**, diagnostic sleep disorder **(C)** and analyzed by RCS analysis between serum cotinine and depression **(D)**, trouble sleeping **(E)**, and diagnostic sleep disorder **(F)**. The X-axis showed the serum cotinine concentration, and the Y-axis showed the estimated risk for depression **(A)**, trouble sleeping **(B)**, diagnostic sleep disorder **(C)** and the estimated odds ratio for depression **(D)**, trouble sleeping **(E)**, and diagnostic sleep disorder **(F)**.

The GAM and RCS models were applied to estimate the nonlinear relationship between serum cotinine concentrations and depression and sleep disorders. As **Figure 2** displayed, the nonlinear relationships between serum cotinine and depression (EDF = 4.507, p<0.001), trouble sleeping (EDF =6.704, p<0.001) and diagnostic sleep disorder (EDF = 2.667, p<0.001) were detected. In addition, RCS models presented inverted L-shape patterns in the associations.

### 3.4 The subgroup associations between serum cotinine and depression and sleep disorders stratified by gender

**Figure 3:**
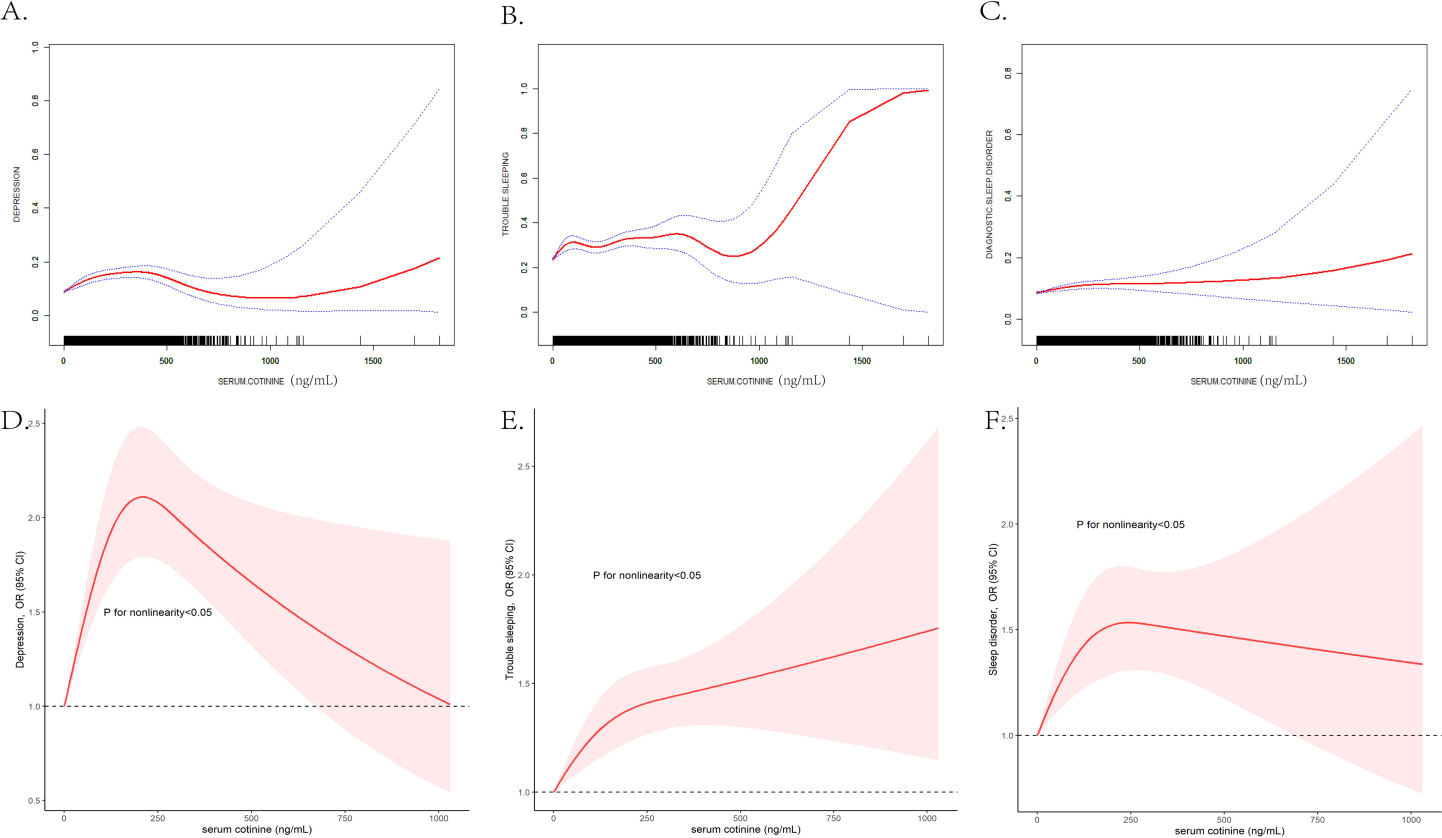
The gender-stratified non-linear association analyzed by the GAM between serum cotinine and depression **(A)**, trouble sleeping **(B)**, diagnostic sleep disorder **(C)**, and analyzed by RCS analysis between serum cotinine and depression **(D)**, trouble sleeping **(E)**, and diagnostic sleep disorder **(F)**. The X-axis showed the serum cotinine concentration, and the Y-axis showed the estimated risk for depression **(A)**, trouble sleeping **(B)**, diagnostic sleep disorder **(C)** and the estimated odds ratio for depression **(D)**, trouble sleeping **(E)**, and diagnostic sleep disorder **(F)**.

The linear relationships between serum cotinine and depression and sleep disorders stratified by gender were listed in **Table 4** (for males) and **Table 5** (for females). The smooth curve fitting methods and RCS curves were presented in **Figure 3**. In male participants, serum cotinine showed positive associations with depression and trouble sleeping (**Table 4**). In female participants, serum cotinine showed positive associations with depression, trouble sleeping, and diagnostic sleep disorder (**Table 5**).

**Table 4:** The relationship between serum cotinine and depression and sleep disorders in male participants.

		Model 1		Model 2	
		OR (95%CI)	*p*	OR (95%CI)	*p*
Depression					
	continuous	1.11 (1.07,1.15)	<0.001	1.05 (1.01,1.09)	0.014
	Q1	ref.		ref.	
	Q2	1.20 (0.81,1.76)	0.351	1.13 (0.68,1.90)	0.623
	Q3	1.84 (1.31,2.58)	<0.001	1.07 (0.65,1.75)	0.799
	Q4	2.85 (2.00,4.07)	<0.001	1.68 (1.07,2.66)	0.027
	P for trend	<0.001		0.020	
Trouble sleeping					
	continuous	1.05 (1.02,1.07)	<0.001	1.04 (1.02,1.07)	<0.001
	Q1	ref.		ref.	
	Q2	1.24 (1.00,1.53)	0.049	1.24 (1.00,1.54)	0.053
	Q3	1.61 (1.30,1.99)	<0.001	1.58 (1.26,1.99)	<0.001
	Q4	1.54 (1.24,1.92)	<0.001	1.58 (1.25,1.99)	<0.001
	P for trend	<0.001		<0.001	
Diagnostic sleep disorder					
	continuous	1.03 (0.99,1.06)	0.122	1.04 (1.00,1.07)	0.065
	Q1	ref.		ref.	
	Q2	0.94 (0.71,1.26)	0.678	0.94 (0.70,1.26)	0.666
	Q3	1.15 (0.83,1.58)	0.394	1.15 (0.83,1.61)	0.388
	Q4	1.15 (0.82,1.61)	0.397	1.28 (0.92,1.79)	0.138
	P for trend	0.292		0.426	

Model 1 was adjusted by age, race.

Model 2, for depression, was adjusted by FIPR, educational level, physical activity, alcohol drinking, diabetes, BMI levels, HEI-2015 and trouble sleeping; for trouble sleeping and diagnostic sleep disorder was adjusted by age, gender, race, FIPR, educational level, physical activity, alcohol drinking, diabetes, BMI levels, HEI-2015 and depression.

**Table 5:** The relationship between serum cotinine and depression and sleep disorders in female participants.

		Model 1		Model 2	
		OR (95%CI)	*p*	OR (95%CI)	*p*
Depression					
	continuous	1.16 (1.14,1.19)	<0.001	1.10 (1.08,1.13)	<0.001
	Q1	ref.		ref.	
	Q2	1.47 (0.99,2.18)	0.059	1.32 (0.87,2.00)	0.185
	Q3	2.17 (1.55,3.03)	<0.001	1.53 (1.08,2.17)	0.018
	Q4	4.70 (3.33,6.62)	<0.001	2.75 (1.88,4.04)	<0.001
	P for trend	<0.001		<0.001	
Trouble sleeping					
	continuous	1.08 (1.06,1.10)	<0.001	1.05 (1.03,1.08)	<0.001
	Q1	ref.		ref.	
	Q2	0.97 (0.8,1.18)	0.779	0.90 (0.73,1.12)	0.335
	Q3	1.27 (0.98,1.63)	0.066	1.09 (0.84,1.43)	0.499
	Q4	1.92 (1.54,2.4)	<0.001	1.47 (1.15,1.88)	0.003
	P for trend	<0.001		<0.001	
Diagnostic sleep disorder					
	continuous	1.07 (1.04,1.10)	<0.001	1.06 (1.02,1.09)	0.003
	Q1	ref.		ref.	
	Q2	1.10 (0.79,1.54)	0.560	0.98 (0.68,1.40)	0.896
	Q3	1.14 (0.83,1.58)	0.405	0.97 (0.69,1.37)	0.873
	Q4	1.88 (1.39,2.56)	<0.001	1.62 (1.16,2.26)	0.006
	P for trend	<0.001		<0.001	

Model 1 was adjusted by age, race.

Model 2, for depression, was adjusted by FIPR, educational level, physical activity, alcohol drinking, diabetes, BMI levels, HEI-2015 and trouble sleeping; for trouble sleeping and diagnostic sleep disorder was adjusted by age, gender, race, FIPR, educational level, physical activity, alcohol drinking, diabetes, BMI levels, HEI-2015 and depression.

The GAM, smooth curve fitting methods and RCS models applied to estimate the dose–response curve of associations between serum cotinine and mental health disorders. As **Figure 3** displayed, in female participants, the nonlinear associations between serum cotinine and depression (EDF = 2.722, p<0.001), trouble sleeping (EDF = 2.512, p<0.001) and diagnostic sleep disorder (EDF = 2.844, p<0.001) were detected; in female participants, the nonlinear associations between serum cotinine and depression (EDF = 3.153, p<0.001) and trouble sleeping (EDF = 7.041, p<0.001) were detected and the association between serum cotinine and of diagnostic sleep disorder was quiet different (EDF = 1.004, p=0.034). In addition, the nonlinear relationships stratified by gender were detected in RCS models, and the trend was different from smooth curve fitting method. In addition, the associations of serum cotinine concentrations with depression, trouble sleeping and diagnostic sleep disorder were stronger in female participants than males.

## 4 Discussion

In the current study, we examined the correlation between serum cotinine levels and depression and sleep disorders in a representative sample of adults in the United States. Our findings revealed that higher serum cotinine levels were associated with an increased risk of depression and sleep disorders (self-reported trouble sleeping and diagnostic sleep disorders). This association was significant in both the total sample and female participants. Additionally, we identified non-linear relationships between serum cotinine levels and depression and sleep disorders and the associations were stronger in females than males.

Current evidence demonstrated that TSE increased risk of depression and sleep disorders. A mendelian randomization study have indicated a causal relationship between smoking including lifetime smoking and smoking initiation, and depression (15). It was remarkably, however, that the association between SHS and depression reported inconsistent results in previous studies, although majority of them reported significant association (6, 35). Self-reported smoking was found to be significantly associated with increased difficulty of initiating sleep (36). Furthermore, a cross-sectional study conducted in Korea found a significant association between urinary cotinine levels and depression symptoms (37). Elevated urinary cotinine levels have also been linked to a higher likelihood of experiencing sleep problems (38). Similarly, our findings indicated that higher TSE is associated with increased risks of depression and sleep disorders.

Furthermore, the non-linear relationships observed in our study suggested the presence of potential inflection points in the effect of serum cotinine on mental health disorders. This finding potentially supported the self-medication hypothesis that smokers may experience withdrawal symptoms and addiction when nicotine levels decreased, leading them to seek more nicotine to alleviate these symptoms (11, 39, 40). The inflection level exceeded 200 ng/mL, that accounts for majority of the cotinine-detectable population in our study. This indicated that individuals with nicotine addiction might reach higher nicotine levels to fulfill their physical and psychological needs, unexpectedly alleviating their symptoms depression and sleep disorders. Additionally, evidence suggested that the association between smoking and depression may be bidirectional, as chronic smokers may attempt to alleviate depressive symptoms by smoking, which can affect sleep architecture (11, 41). In general, from a public health perspective, controlling TSE is more beneficial to people’s health, especially for those who are passively exposed to tobacco (generally considered to have serum cotinine levels below 10 ng/mL), as it can reduce the risk of depression and sleep disorders.

Interestingly, our results showed that females had lower serum cotinine levels but a higher prevalence of depression and trouble sleeping, along with stronger associations between serum cotinine levels and both depression and sleep disorders. Previous studies on gender differences in TSE and depression and sleep disorders have yielded inconsistent findings. Park et al. reported a significant association between urinary cotinine levels and depression symptoms only in females (37). A meta-analysis including 24 studies revealed no gender difference in the association between SHS and depressive symptoms (42). Zandy et al. (38) reported stronger associations of TSE and sleep problems in females, while Mehari (36) and Ioannidou (43) et al. reported no gender differences. These differences might be caused by the different diagnosis of smoking status, criteria or types of depressive systems and sleep disorders, and study population. The gender difference may be explained by the fact that females are more likely to develop mental disorders and are more vulnerable to TSE. Previous studies have suggested that ovarian hormones could affect the hypothalamic-pituitary-adrenal (HPA) axis, dopaminergic, noradrenergic, and gamma-aminobutyric acid (GABA) systems, which are also the targets of nicotine (44, 45). Estrogen could accelerate nicotine metabolism (46). In addition, females are more susceptible to the effects of nicotine on neuro-steroid production due to the menstrual cycle phase and sex hormones (40, 47). These enhance the women’s sensitivity to nicotine’s rewarding and reinforcing effects, making women more vulnerable to TSE. Moreover, several social psychological theories propose explanations for the gender gap in depression (48, 49), demonstrating that females were more vulnerable to nicotine dependence due to their responses to stress and negative mood (40, 50, 51). Taken together, these factors contributed to the increased susceptibility of females to TSE.

Our study had several clear strengths. First, different from the evaluation of smoking patterns in previous studies, serum cotinine concentrations were used as a more objective and accurate biomarker to reflect TSE in this study. Second, to the best of our knowledge, this study was the first to systematically investigate the associations between cotinine levels and depression and sleep disorders in the general US population, providing new epidemiological evidence on the impact of tobacco smoke on mental health. Third, we accounted for complex sampling weights in our analysis, which was conducted using data from four cycles of the NHANES program and involved a large sample size, thereby increasing the robustness of our findings. Finally, the use of smooth curve fitting methods and restricted cubic spline models allowed us to identify non-linear relationships between serum cotinine levels and mental health disorders, providing a deeper understanding of the risks associated with TSE. However, it should be noted that the differences observed in the two non-linear relationship models may be due to variations in the equations used in the gender-stratified models.

Some limitations in our study should be noted. First, NHANES was a cross-sectional survey so that the temporal relationship between exposure and outcome was uncertain. Second, the definitions of depression in our study defined by PHQ-9, and sleep disorders (trouble sleeping and sleep disorders) were verified by questionnaire survey method, which may cause diagnosis bias. It seemed limited to classify sleep disorders as mental health issues, but there were studies that reported sleep disorder as mental health (6, 52). Third, the study population with serum cotinine concentrations above LLOD was from NHANES program and the generalization of our conclusion to other population should be cautious. Lastly, although we accounted for numerous covariates in our model, there remains the possibility that unmeasured variables, such as genetic factors, medication use, and occupational habits, could influence our findings.

## 5 Conclusion

In this study, we detected nonlinearity relationships between serum cotinine and depression and sleep disorders. The results suggested that TSE was an independent the risk of depression and sleep disorders and there was stronger association in females than males. Our findings provide novel epidemiological evidence about direct and/or secondhand tobacco smoke exposure affects the mental health condition of the general US population, suggesting that prohibiting TSE in public spaces is beneficial to public health. The causal relationship and potential mechanisms of the sex-difference relation between TSE and mental health need to be further investigated.

## Data Availability

Publicly available datasets were analyzed in this study. This data can be found here: https://www.cdc.gov/nchs/nhanes/index.htm.
