# Supplementary material for: Sex-specific associations of serum cotinine levels with depressive symptoms and sleep disorders in American adults: NHANES 2007-2014
Source: Front Psychiatry. 2024 Dec 10;15:1434116. doi: 10.3389/fpsyt.2024.1434116 (PMC11666554; doi:10.3389/fpsyt.2024.1434116)
Supplement: Supplementary file 1 [file Table1.docx]

|  | **Total (N=12599)** | **Male (N=6611)** | **Female (N=5988)** | **p** |
| --- | --- | --- | --- | --- |
| **Age** | 45.49±16.45 | 44.91±16.13 | 46.13±16.78 | <0.001 |
| **Race, n (weighted %)** |  |  |  | <0.001 |
| Mexican American | 2777(12.6) | 1494(13.9) | 1283 (11.2) |  |
| Non-Hispanic White | 5804 (68.2) | 3070 (68.5) | 2734 (67.9) |  |
| Non-Hispanic Black | 2920 (12.4) | 1455 (10.9) | 1465 (14.1) |  |
| Other or multiracial | 1908 (6.7) | 592 (6.7) | 506 (6.7) |  |
| **FIPR, n (weighted %)** |  |  |  | <0.001 |
| =<1.30 | 4521 (25.2) | 2198 (22.9) | 2323 (27.9) |  |
| 1.30-3.50 | 4608 (36.2) | 2422 (35.4) | 2186 (37.0) |  |
| >3.50 | 3470 (38.6) | 1990 (41.8) | 1479 (35.1) |  |
| **Educational levels, n (weighted %)** |  |  |  | <0.001 |
| Less than high school graduated | 3276 (18.5) | 1777 (19.0) | 1499 (18.0) |  |
| High school graduated | 3145 (24.9) | 1741 (26.4) | 1404 (23.2) |  |
| Some college or AA degree | 3775 (32.8) | 1785 (30.3) | 1990 (35.7) |  |
| College graduate or above | 2403 (23.7) | 1308 (24.4) | 1095 (23.1) |  |
| **Alcohol drinking, n (weighted %)** |  |  |  | <0.001 |
| No | 3076 (19.6) | 960 (11.8) | 2116 (28.1) |  |
| Yes | 9523 (80.4) | 5651 (88.2) | 3872 (71.9) |  |
| **Serum cotinine (median [IQR])** | 0.14(0.03, 128.00) | 0.27(0.04, 165.37) | 0.09(0.03, 73.30) | <0.001 |
| **Diabetes, n (weighted %)** | 2247 (13.5) | 1213 (13.6) | 1034 (13.5) | 0.894 |
| **BMI levels, n (weighted %)** |  |  |  | <0.001 |
| <25 | 3640 (29.9) | 1876 (27.1) | 1764 (33.1) |  |
| 25 to <30 | 4118 (33.0) | 2456 (37.6) | 1662 (27.9) |  |
| ≥30 | 4841 (37.1) | 2279 (35.3) | 2562 (39.0) |  |
| **Physical activity** |  |  |  | <0.001 |
| No | 3240 (22.0) | 1387 (17.6) | 1853 (26.7) |  |
| Low | 5231 (43.2) | 2489 (38.3) | 2742 (48.6) |  |
| High | 4128 (34.8) | 2735 (44.0) | 1393 (24.7) |  |
| **HEI-2015** | 52.09 ±13.08 | 50.91±12.65 | 53.39 ±13.40 | <0.001 |
| **Depression, n (weighted %)** | 1295 (9.0) | 471(6.2) | 824 (12.0) | <0.001 |
| **Trouble sleeping, n (weighted %)** | 3245 (27.0) | 1418 (22.5) | 1836 (32.0) | <0.001 |
| **Diagnostic sleep disorder, n (weighted %)** | 1152 (9.3) | 610 (9.5) | 542 (9.1) | 0.605 |

# Supplementary materials

**Table S1 basic characteristic of study population stratified by gender**

Sampling weights were applied for calculation of demographic descriptive statistics. The p value was calculated after weighting.

Abbreviations: FIPR, family income-to-poverty index; BMI, body mass index; HEI-2015, healthy eating index 2015.
